# Gastric cancer in patients with gastric atrophy and intestinal metaplasia: A systematic review and meta-analysis

**DOI:** 10.1371/journal.pone.0219865

**Published:** 2019-07-26

**Authors:** Maryam Akbari, Reza Tabrizi, Sina Kardeh, Kamran B. Lankarani

**Affiliations:** 1 Health Policy Research Center, Institute of Health, Student Research Committee, Shiraz University of Medical Sciences, Shiraz, Iran; 2 Cellular and Molecular Medicine Student Research Group, Shiraz School of Medicine, Shiraz, Iran; 3 Health Policy Research Center, Institute of Health, Shiraz University of Medical Sciences, Shiraz, Iran; University of Mississippi Medical Center, UNITED STATES

## Abstract

**Aim:**

Intestinal metaplasia (IM) and gastric atrophy (GA) are precancerous lesions in the stomach. There is a large debate on natural course of these lesions and surveillance strategy in these patients. This meta-analysis was aimed to find the most appropriate follow up and the rate of progression from IM and GA to GC.

**Methods:**

This meta-analysis is followed and reported according to the Preferred Reporting Items for Systematic Reviews and Meta-Analyses (PRISMA) guidelines. Electronic databases including EMBASE, PubMed, Web of Science databases, Scopus, and the Cochrane Library were searched until July 2018. Cochran’s Q test and I-square (I^2^) test were used to examine heterogeneity across included studies. We pooled data using random-effect or fixed effect models indicated as incidence rate or proportion with 95% confidence intervals (CI). The variables of study included demographic data, endoscopy interval, follow up interval and time, GA and IM type and GC stage. Moreover, incidence rate of GC and progress rate, regress and persistence proportion in both GA and IM patients were assessed.

**Results:**

Overall, 68 original articles out of 32981 citations were included in our meta-analysis. The pooled GC incidence rate in patients with GA was 1.24 (95% CI, 0.80, 1.76; I^2^: 83.6%) cases per 1,000 person-years. The rates of later diagnosis of IM and gastric dysplasia in patients with GA were estimated as 41.42 (95% CI, 3.11, 64.45; I^2^: 95.6%) and 6.23 (95% CI, 2.34, 11.46; I^2^: 83.0%) cases per 1,000 person-years, respectively. The pooled regressed proportion was 32.23 (95% CI, 18.07–48.02; I^2^: 94.0%) and the persistence proportion was 38.83 (95% CI, 20.20–59.13; I^2^: 97.0%) per 100 observations in GA patients. In IM studies, the pooled incidence rate of GC was 3.38 (95% CI, 2.13, 4.85; I^2^: 93.4%) cases per 1,000 person-years. The progressed rate to dysplasia in IM patient was estimated to be 12.51 (95% CI, 5.45, 22.03; I^2^: 95.1%) cases per 1,000 person-years. The pooled regressed proportion was 31.83 (95% CI, 25.48–38.51; I^2^: 91.0%) and the persistence proportion was 43.46 (95% CI, 32.52–54.71; I^2^: 96.0%) per 100 observations in IM patients.

**Conclusion:**

Overall, the incidence of GC in patients with IM and GA are low but there is heterogeneity in data with the highest rate in Asian, males with those with incomplete IM. There is probability of regression or persistence without progression in patients with IM and GA who receive appropriate management.

## Introduction

Gastric cancer remains among the top 5 most frequently diagnosed cancer and is the third leading cause of cancer mortality worldwide and is responsible for over 1,000,000 new cases in 2018 and an estimated 783,000 deaths [1]. Despite decrease in incidence, gastric cancer (GC) is still one of the most lethal malignancies worldwide [2, 3].

Given that early cancers are usually asymptomatic, two-thirds of the patients present at an advanced stage when curative resection is not possible. Despite the advances in treatment, the prognosis of gastric cancer varies greatly depending on stages. While the 5-year survival rates for advanced gastric cancer are dismal and less than 30% in USA and Europe, there is 5-year survival rate over 90 to 95% in Japan most probably due to complemented strategies for detection of GC in the early stages [4, 5].

This geographical variation is due to high incidence in East Asia and introduction of surveillance programs. On the other hand, setting up such programs has been impacted in USA and Europe by the lower incidence of *Helicobacter pylori* (*H*. *pylori*) [6, 7].

*Helicobacter pylori (H*.*pylori)* is the most common contributing factor to GC and has been classified as a class I carcinogen as it is a conspicuous role player in promotion of gastric inflammation [8, 9].

Sequential changes of the gastric mucosa including loss of structured glandular cells and replacement with intestinal-type epithelium, pyloric-type glands are believed to be a multistep process initiated by chronic inflammation secondary to *H*.*pylori* infection with resultant GA and IM as premalignant lesions [10, 11].

Therefore, *H*. *pylori* is of utmost importance as both GA and IM are postulated to predispose to GC through higher risk of genetic instability [12–15].

There are several classifications of IM, based on the histology and types of mucin secreted. Currently, they are generally subclassified as “complete” (small intestinal type) or “incomplete” (colonic type, which has the highest risk to progress to GC) [16].

Although there is a recent debate on whether cancer cells develop directly from intestinal metaplastic cells or IM is a surrogate marker of cancer, the presence of this tissue transformation has been associated with later development of GC in epidemiologic studies [17]. This produces a window of opportunity for screening and early detection of GC [18].

Although there was insufficient evidence of mortality reduction from GC when South Korea introduced endoscopic screening as the first country in a national program, increasingly accumulated evidence over the last decade regarding the effectiveness of endoscopic surveillance for GC has been obtained from new research results [19, 20]. Nevertheless, quality assurance and protocol design need to be established, as there are wide variations of the proposed screening methods and timing after detection of GA or IM and most of them are based on expert view rather than on through evidence.

In this study, we scrutinized the available data through systematic review and meta-analysis to find the rate of progression from IM and GA to GC. This may be of help to develop more evidence-based strategies in follow up of patients with IM and GA and in screening for GC.

## Materials and methods

### Search strategy and study selection

This study is followed and reported according to the Preferred Reporting Items for Systematic Reviews and Meta-Analyses (PRISMA) guidelines.

Original studies reporting the incidence rate of gastric cancer in patients with gastric atrophy or intestinal metaplasia were identified by two authors (MA and RT) who independently searched online databases including EMBASE, PubMed, Web of Science databases, Scopus, Cochrane Library, and ongoing projects until July 2018.

The following keywords and MeSH terms were used for online searches; ["intestinal" or "metaplasia" or "atrophic" or "atrophy"] AND ["gastritis" or "gastric" or "stomach neoplasms" or "stomach" or "esophagogastric junction"] AND ["cancer" or "tumor" or "neoplasm" or "carcinoma" or "adenocarcinoma").

Reference lists of suitable studies and related previous review articles were checked manually to increase search sensitivity and identify any related study. Searches were restricted to original studies published in English language among humans.

### Inclusion and exclusion criteria

Two independent authors (MA and RT) included studies that met the following inclusion criteria: natural history studies as original observational cohort either with prospective or retrospective design and also interventional studies, performed on adult humans, contained sufficient data for calculating the incidence rate and 95% confidence intervals (CI) of gastric cancer in patients with GA or IM. Other study types including *in vitro* studies, animal experiments, case report, case series, seminar abstracts without full texts or the protocol of studies without results, same population of other studies, and studies that had a follow-up period of less than 6 months were excluded from the current meta-analysis. The value of weighted kappa test was 92% between author agreements during the systematic searches. Any case of discrepancies was resolved by consensus or discussion with a third author (K-BL).

### Data extraction and quality assessment

Two investigators (MA and RT) individually extracted the required information from each included study using a standard abstraction form in excel sheet. The following data were extracted: first author’s name, year of publication, location of study, the demographic characteristics of participants, study design, sample size (in both GA or IM groups), follow-up time, number of endoscopies in study duration, number of GC cases, type of GC (early or advanced), and frequency of regress and progress to other stages in participants with GA or MI.

The modified version of the Newcastle-Ottawa Scale was used to assess the quality of included primary studies to our meta-analysis [1]. This scale used the following criteria for quality assessment: "representativeness of the exposed study, ascertainment of exposure, demonstration that outcome of interest was not present at start of study, assessment of outcome, was follow-up long enough for outcomes to occur, adequacy of follow up of studies". Therefore, modified version of the Newcastle-Ottawa's total score lower than 3 represented the low quality, 3–4 indicated moderate quality, and maximum score 5–6 showed high quality of included studies. Any disagreement in extracted data or scored quality of studies between authors was resolved through consensus until discussion with a third author (K-BL).

### Data synthesis and statistical analysis

All statistical analyses were conducted using a meta package in R software (Version 3.3, Statistics Department of the University of Auckland). The metarate function was applied to pool the incidence rate of GC or progressed at each stage to other stages per 1,000 person-years in GA or IM cases. Where the incidence rates were not reported, person-years were estimated using the number of events multiplied by mean or median or midpoint follow-up in years. The metaprop function was used to describe the pooled proportions of regressed and persistence in GA and IM cases per 100 observations. By using metarate and metaprop functions, original studies with incidence rate of 0 person-years or proportion of 0 and 100% were also included in our meta-analysis. Moreover, pooled effect sizes and related CIs were always within admissible values [21]. Heterogeneity across selected studies was assessed using Cochrane’s Q test and I-square (I^2^) statistic. I^2^ > 50% with p < 0.05 showed significant heterogeneity between the studies, and authors used the random effects model with inverse variance method in Freeman-Tukey Double arcsine transformations (IRFT) and Freeman-Tukey Double arcsine transformations (PFT) to pool the incidence rates and proportions; otherwise, the fixed-effect model was used. Because of the existence of higher heterogeneity across selected studies, sensitivity and subgroup analysis were applied as additional analyses. Sensitivity analyses were performed using the leave-one-out method to estimate the impact of one by one included study on reliability of the summary effect sizes.

Subgroup analyses were conducted to examine the source of heterogeneity by the following potential moderator variables: country/region (Asia vs. Europe vs. others), study design (prospective cohort vs. retrospective cohort vs. RCT), male (% study population) (<40% vs. 40–50% vs. ≥50%, NR % male), Age (mean) (≤ 50 years vs. > 50 years vs. NR), type GC (advanced vs. early vs. undetermined), type GA (GA mild vs. GA moderate vs. GA severe vs. undetermined), type IM (IM complete vs. IM incomplete vs. undetermined), follow-up time (< 3 years vs. > 5 years vs. 3–5 years), endoscopy interval (≤ 1 year vs. > 3 years vs. 2–3 years vs. NR) and the Newcastle-Ottawa's overall scores (moderate vs. high quality). We also conducted meta-regressions to assess whether potential covariates including the number of participant in study, the year of study could explain the heterogeneity across studies. Publication bias was evaluated visually and statistically using funnel plot symmetry tests and using quantitative Begg (rank correlation) and Egger (linear regression) tests in our meta-analysis. The trim and fill method was applied to estimate an adjusted pooled ES (95% CI) if publication bias existed across included studies. P<0.05 was considered statistically significant.

## Results

### Search results

**Fig 1:**
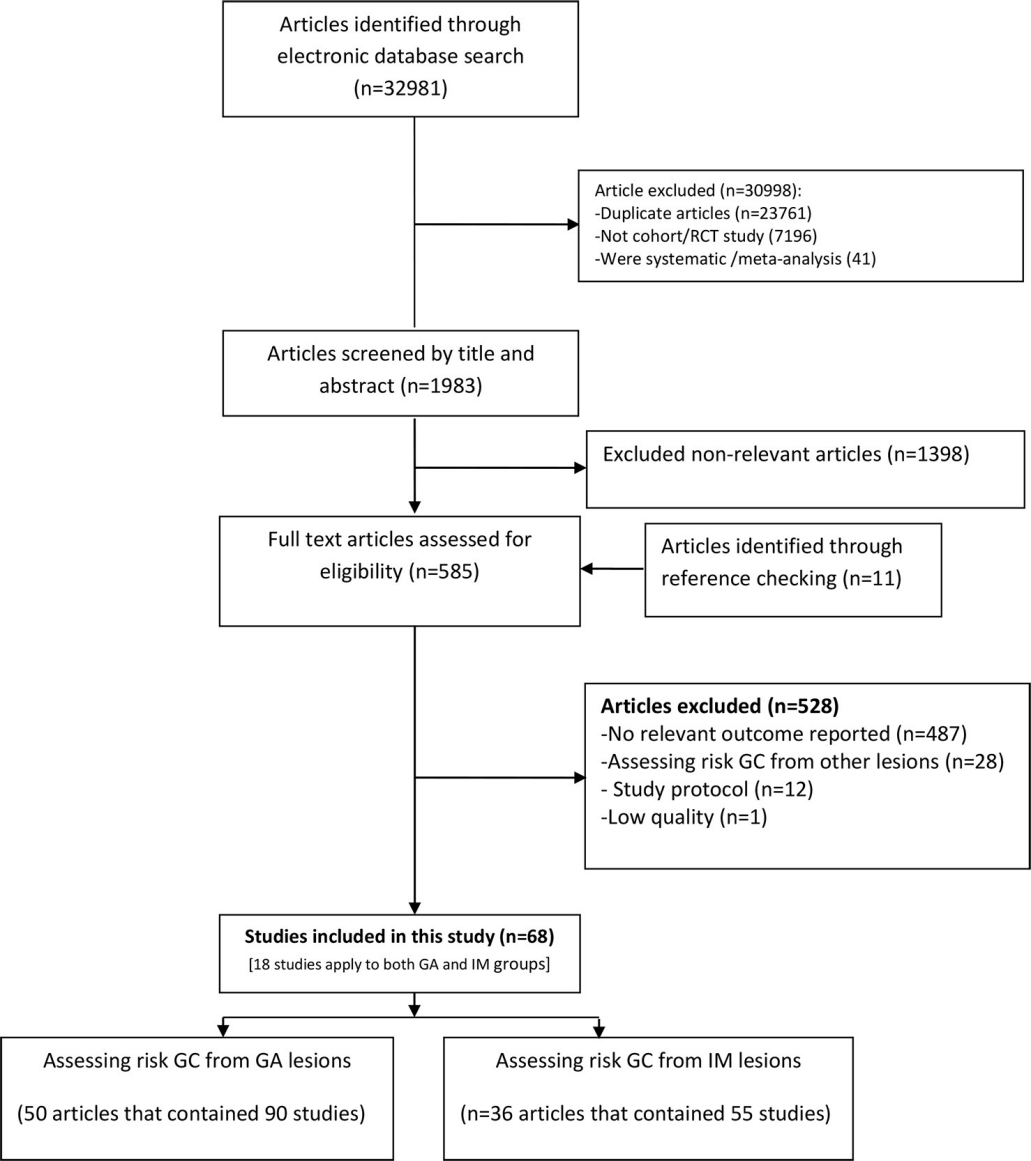
Literature search and review flowchart for selection of studies.

As indicated by the step by step screening process in **Fig 1**, finally 68 articles [22–88] out of 32981 reports were included after removing the duplicates, reviewing the title and abstracts, and assessing the eligibility of identified articles based on our inclusion criteria.

Forty one [22–62] were alphabetically included several times because each of these articles contained information about the GC incidence rate based on various types of metaplasia (GA and/or IM) among the same study participants. Sixty-eight original articles containing 101317 participants were included in the current meta-analysis. Fifty articles have reported assessing the incidence rate of GC on GA lesions and 36 articles on IM lesions. The selected articles were published between 1960 and 2018. Seven of the 68 citations included in our review were conducted in Japan, 8 in Finland, 7 in China and USA, 6 in Italy, 4 in Netherlands, 3 in UK, 2 in Korea, London, and Spain, 1 in Australia, Chile, Colombia, London, Portugal, Scotland, Slovenia, Sweden, Taiwan, and Thailand. The remaining one was conducted within several countries as a multicenter study [57]. The detailed characteristics of the selected articles are summarized in **Table 1**.

**Table 1:** Characteristics of included studies.

First Author	Publication year	Non-cohort/ Non-GC	Country/Study design	Male (%)	Mean age (years)	Endoscopy Interval	Follow-up [mean/median/mid-point] (years)	Study quality (score)
**Zhang et al. [72]**	2018	332/16	China/ Retrospective cohort	52	63	1	9.17	6
**Mera (a, b) et al. [29]**	2018	649/7	USA/ RCT	46	51.9	3	8	5
**Hollander (a, b) et al. [35]**	2018	11/0	Netherlands/ prospective cohort	49	57.9	2	4.7	6
**Song et al. [73]**	2017	3714/37	Korea/ Retrospective cohort	64.62	47.81	2	6.88	5
**Pittayanon (a,b) et al. [37]**	2017	81/0	Thailand/ Retrospective cohort	51	63	1	4.05	4
**Gomez et al. [75]**	2017	468/21	USA/ Retrospective cohort	44.4	61	3	3	4
**Toyoshima (a,b,c,d,e,f) et al. [44]**	2017	68/0	Japan/ Retrospective cohort	46.34	54.1	1	2.46	5
**Gonzalez(a,b,c) et al. [22]**	2016	219/15	Spain/ Retrospective cohort	57.53	53.19	18	12	5
**Li et al. [70]**	2016	4146/37	United States/ Retrospective cohort	48	66	NR	7.1	5
**Reddy et al. [71]**	2016	923/8	United States/ Retrospective cohort	NR	68	3	4.6	4
**Shichijo (a,b,c,d,e,f) et al. [40]**	2016	399/6	Japan/ Retrospective cohort	56	58.4	1	6.2	4
**Vohlonen et al. [87]**	2016	606/8	Finland/ prospective cohort	100	57.5	17	8.5	5
**Lahner et al.[63]**	2015	200/4	Italy/ prospective cohort	33	55	4	7.5	4
**Song (a,b) et al. [24]**	2015	14285/116	Sweden/ Retrospective cohort	55	60.3	NR	10.1	5
**Horsley-Silva et al. [69]**	2015	200/14	United States/ Retrospective cohort	50	68	NR	4.1	3
**Take (a,b) et al. [38]**	2015	192/9	Japan/ prospective cohort	91.14	48.9	1	10.4	6
**Shichijo (a,b,c) et al. [41]**	2015	497/7	Japan/ Retrospective cohort	51	60.1	1	6.7	4
**Sakitani (a,b,c,d,e,f) et al. [45]**	2015	215/3	Japan/ Retrospective cohort	81.3	64.8	1	4.78	5
**Zhou et al. [85]**	2015	266/9	China/ Retrospective cohort	NR	NR	5	2.5	5
**Adgey et al.[88]**	2014	29/1	United Kingdom/ prospective cohort	45	62	1	4.7	4
**Bleibe et al.[67]**	2013	675/14	United States/ Retrospective cohort	49	61	NR	5.3	3
**Wong (a,b) et al. [62]**	2012	65/0	China/ RCT	46.5	52.9	1	1	5
**Miki (a,b,c,d,e) et al. [30]**	2011	33/4	Japan/ Retrospective cohort	NR	49.3	1	9.3	6
**Take (a,b,c) et al.** **[46]**	2011	511/1	Japan/ prospective cohort	86.7	50.8	1	7	5
**Vannella et al. [66]**	2010	300/3	Italy/ prospective cohort	32	54	2	4.3	5
**De Vries et al. [68]**	2010	101/0	Netherlands/ prospective cohort	50	60.7	1	2.3	5
**Mizuno (a,b) et al. [48]**	2010	69/3	Japan/ prospective cohort	35.36	NR	NR	9.3	4
**Gonzalez (a,b,c) et al. [52]**	2010	88/16	Spain/ prospective cohort	54.5	56.5	1	12.8	5
**Yanaoka (a,b) et al. [51]**	2009	1329/30	Japan/ prospective cohort	100	50.4	1	9.5	6
**Sun (a,b,c) et al. [59]**	2009	19/0	China/ prospective cohort	66.2	50	4	7	5
**Kim et al. [69]**	2008	515/4	Korea/ prospective cohort	88	45	3	10.2	4
**DE Varies (a,b) et al. [47]**	2008	61707/874	Netherlands/ prospective cohort	52.5	66.5	2	2.8	5
**Takata et al. [65]**	2007	101/8	Japan/ prospective cohort	58	56	1	5.2	4
**Take (a,b,c) et al. [49]**	2007	316/0	Japan/ prospective cohort	89.47	49.9	1	3.9	4
**Tava et al. [79]**	2006	259/4	Italy/ prospective cohort	51	60	2	3.9	4
**Watabe (a, b) et al. [31]**	2005	1082/18	Japan/ prospective cohort	65.89	52	1	4.7	4
**Lahner (a,b,c) et al. [42]**	2005	38/1	Italy/ prospective cohort	31.6	51	2	3	4
**Kamada et al. [78]**	2005	453/2	Japan/ prospective cohort	64	62.4	1	3.9	4
**Leung (b,c) et al. [26]**	2004	4/0	China/ RCT	NR	52	5	5	4
**Ohata (a, b) et al. [32]**	2004	1316/24	Japan/ prospective cohort	NR	49.8	1	5	4
**Dinis-Ribeiro (a,b,c,d) et al. [36]**	2004	58/0	Portugal/ Retrospective cohort	48	55	2	12.5	5
**Wong (a,b) et al. [61]**	2004	57/0	China/ RCT	54.1	42.4	0.6	4	5
**Whiting (a, b) et al. [28]**	2002	11/2	United Kingdom/ prospective cohort	NR	> 40	1	8.6	4
**Kokkola (a,b) et al. [60]**	2002	13/0	Finland/ prospective cohort	100	62	NR	2.5	4
**Uemura (a,b,c,d) et al. [50]**	2001	381/3	Japan/ prospective cohort	57.3	52.3	3	7.6	4
**El Zimaity (a,b) et al. [53]**	2001	34/0	USA/ Retrospective cohort	93.6	60	1	4.5	4
**Lin (a,b,c) et al. [54]**	2001	15/14	Taiwan/ prospective cohort	NR	NR	1	10	3
**Inoue (a,b,c) et al. [23]**	2000	1356/47	Japan/ prospective cohort	48	50.7	1	10	4
**Klinkenberg-knol (a,b,c) et al. [57]**	2000	9/0	Multicenter (N, A, C, G)/ prospective cohort	57.3	64	1	6.5	4
**You (a,b,c,d) et al. [43]**	1999	1032/1	China/ prospective cohort	51.63	45.5	2	4.5	4
**Valle et al. [84]**	1996	13/0	Finland/ Retrospective cohort	50	63	31	15.5	4
**Kuipers(a,b,e,d) et al. [56]**	1995	5/0	Netherlands/ prospective cohort	NR	48	NR	11.5	4
**Filipe (a,b,c) et al. [33]**	1994	197/5	Slovenia/ Retrospective cohort	73	NR	5	9.5	4
**Tatsuta (a,b,c) et al. [25]**	1993	246/5	Japan/ Retrospective cohort	80	NR	8	19	4
**Rokkas et al. [74]**	1991	26/11	London/ Retrospective cohort	NR	NR	1	5	4
**Correa (b, c) et al. [27]**	1990	298/1	Colombia/ prospective cohort	NR	NR	5	5.1	4
**Ramesar (a,b,c) et al. [55]**	1987	16/0	Scotland/ Retrospective cohort	50	66.9	NR	6.5	4
**Testoni (a,b) et al. [58]**	1987	166/7	Italy/ prospective cohort	NR	54	1	9	4
**Ectors et al. [77]**	1986	90/1	United Kingdom/ Retrospective cohort	NR	NR	NR	4.5	4
**Filipe (a,b) et al. [34]**	1985	232/11	London/ prospective cohort	16	56.8	NR	0.5	4
**Ihamauki (a,b) et al. [39]**	1985	116/10	Finland/ prospective cohort	NR	NR	8	13	4
**Siurala et al. [64]**	1974	116/10	Finland/ prospective cohort	NR	64	NR	21	4
**Cheli et al. [80]**	1973	65/9	Italy/ prospective cohort	NR	48.5	7	3.5	4
**Aste et al. [83]**	1973	36/0	Chile/ prospective cohort	69.4	41.5	NR	5.6	3
**Walker et al. [76]**	1971	40/4	Australia/ Retrospective cohort	NR	53.75	NR	15	4
**Siurala et al. [81]**	1966	116/9	Finland/ prospective cohort	NR	NR	6.5	7	4
**Siurala et al. [86]**	1961	39/1	Finland/ prospective cohort	55.5	52	8	6	4
**Siurala et al. [82]**	1960	53/5	Finland/ prospective cohort	58.4	50.8	1	5.8	4

NR, not reported; GC, gastric cancer

Subscript letters (a,b,c, …) shows different types of precancerous lesions for estimating the relevant outcomes. We treated each type of lesions in these articles as a separate study to be included in the current meta-analysis.

### Incidence rate of GC and progress rate, regress and persistence proportion in GA patients

Fifty articles involving 90 studies were included, and a total number of 68893 patients in the GA studies, 920 GC cases were found over the follow-up period from 1 year to 21 years (a total of 454679.2 person-years). The pooled incidence rate of GC in patients with GA was 1.24 (95% CI, 0.80, 1.76; I^2^: 83.6%) cases per 1,000 person-years with random-effects model (**Table 2)**.

**Table 2:** The estimation of GC incidence rate in patients with GA based on subgroup analyses.

Variables			K	I^2^ (%)	Q test	Inc rate per 1,000 (95% CI)	Test for subgroup differences p-value*
**GA**							
	**Total**		90	83.6	541.63	1.24 (0.80, 1.76)	-
	**Region**						
		Asia	56	77.4	243.10	2.25 (1.67, 2.90)	0.454
		Europe	28	84.0	170.56	0.74 (0.13, 1.71)	
		Other	6	66.3	14.82	0.00 (0.00, 0.012)	
	**Study design**						
		Prospective cohort	54	76.1	221.89	0.94 (0.48, 1.50)	<0.0001
		Retrospective cohort	32	85.4	211.88	2.72 (1.79, 3.81)	
		Prospective RCT	4	0.0	2.10	0.00 (0.00, 0.001)	
	**Type GA**						
		Undetermined	46	86.6	335.18	1.57 (0.91, 2.34)	<0.0001
		GA mild	16	50.3	30.21	0.00 (0.00, 0.01)	
		GA moderate	14	21.5	16.55	0.94 (0.47, 1.52)	
		GA severe	14	69.1	42.02	4.82 (2.66, 7.48)	
	**Male (% study population)**						
		< 40%	7	0.0	2.64	1.60 (0.79, 2.63)	0.010
		40–50%	16	75.9	62.24	0.02 (0.00, 0.64)	
		≥ 50%	45	85.8	309.52	1.48 (0.91, 2.14)	
		NR	22	75.1	84.51	2.84 (1.35, 4.70)	
	**Age (mean, years)**						
		≤ 50	21	77.7	89.68	1.31 (0.52, 2.34)	0.175
		> 50	58	84.5	368.43	0.94 (0.45, 1.56)	
		NR	11	80.2	50.62	3.59 (1.67, 6.09)	
	**Stage GC**						
		Advanced stage	38	81.4	198.55	3.27 (2.53, 4.10)	0.003
		Early stage	12	78.8	51.94	3.26 (1.37, 5.71)	
		Undetermined	40	75.8	161.48	0.00 (0.00, 1.10)	
	**Follow-up time**						
		< 3 years	12	49.5	21.78	1.36 (0.00, 5.16)	0.330
		> 5 years	51	86.8	377.86	1.63 (1.02, 2.33)	
		3–5 years	27	74.6	102.47	1.14 (0.43, 2.09)	
	**Endoscopy interval**						
		≤ 1 year	45	68.8	141.11	1.89 (1.23, 2.65)	0.053
		> 3 years	15	82.9	103.70	1.71 (0.24, 4.03)	
		2–3 years	19	82.6	103.70	0.74 (0.19, 1.52)	
		NR	11	86.7	75.32	0.26 (0.00, 2.32)	
	**Study quality**						
		high	38	85.1	247.72	1.63 (1.07, 2.85)	0.01
		moderate	52	74.6	200.81	1.20 (0.56, 2.02)	

*Test for subgroup differences (random effects model)

K, number of study; Inc, incidence; GA, gastric atrophy; GC, gastric cancer.

The progressed rates to IM and dysplasia in GA patients were estimated as 41.42 (95% CI, 3.11, 64.45; I^2^: 95.6%) and 6.23 (95% CI, 2.34, 11.46; I^2^: 83.0%) cases per 1,000 person-years in the pooled 9 and 11 studies, respectively. The pooled results of the 10 studies using random-effect model estimated that the regressed proportion was 32.23 (95% CI, 18.07–48.02; I^2^: 94.0%) and the persistence proportion was 38.83 (95% CI, 20.20–59.13; I^2^: 97.0%) per 100 observations among patients with GA (**S1 Table**).

**Fig 2:**
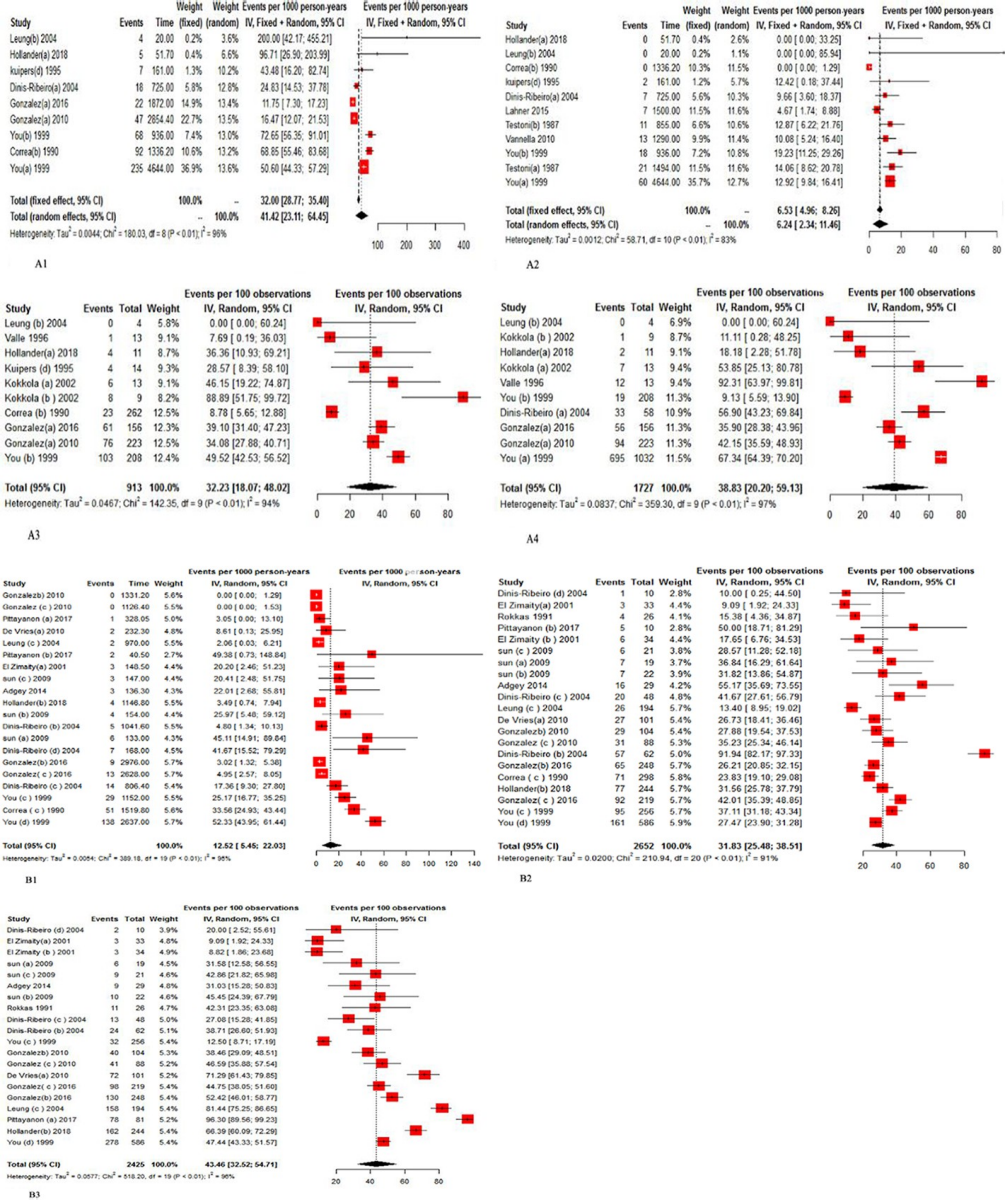
**A-B**. Random effects meta-analysis for (A1) progress rate to IM, (A2) progress rate to Dys, (A3) regress proportion, (A4) persistence proportion in GA patients, and for (B1) progress rate to Dys, (B2) regress proportion, (B3) persistence proportion in IM patients with 95% CI.

Forest plots estimating the progress rates, regress, and persistence proportions in GA patients are shown in **Fig 2**.

### Incidence rate of GC and progress rate, regress and persistence proportion in IM patients

In IM studies, there were a total number of 87326 individuals where 1325 progressed to GC during a follow-up time from 6 months to 16.8 years (a total of 358317.55 person-years). The pooled results of the 55 studies using random-effects model indicated that the incidence rate of GC in IM patients was 3.38 (95% CI, 2.13, 4.85; I^2^: 93.4%) cases per 1,000 person-years (**Table 3**).

**Table 3:** The estimation of GC incidence rate in patients with IM based on subgroup analyses.

Variables			K	I^2^ (%)	Q test	Inc rate per 1,000 (95% CI)	Test for subgroup differences p-value*
**IM**							
	**Total**		55	93.4	819.59	3.38 (2.13, 4.85)	-
	**Region**							
		Asia	21	87.1	155.64	7.58 (4.10, 11.91)	0.029
		Europe	25	95.5	535.79	1.72 (0.36, 3.70)	
		Other	9	89.6	77.00	2.92 (0.97, 5.69)	
	**Study design**							
		Prospective cohort	23	89.1	201.46	4.62 (2.01, 7.99)	0.08
		Retrospective cohort	28	89.0	248.18	3.02 (1.68, 4.64)	
		Prospective RCT	4	87.2	23.46	3.94 (0.00, 13.00)	
	**Type IM**							
		Undetermined	24	96.1	590.09	3.32 (1.82, 5.16)	0.11
		IM complete	13	85.9	85.38	3.24 (0.12, 9.01)	
		IM incomplete	18	87.0	130.64	6.60 (2.73, 11.75)	
	**Male (% study population)**							
		< 40%	2	89.5	9.54	282.17 (0.00, 1135.86)	<0.0001
		40–50%	11	85.0	66.53	0.86 (0.13, 2.01)	
		≥ 50%	33	80.6	164.55	3.84 (2.57, 5.31)	
		NR	9	92.9	112.13	18.10 (5.56, 36.58)	
	**Age (mean, years)**							
		≤ 50	9	80.0	40.06	3.21 (0.25, 8.28)	0.037
		> 50	37	94.5	655.01	2.30 (1.08, 3.85)	
		NR	9	93.4	122.08	14.04 (5.88, 25.27)	
	**Stage GC**							
		Advanced stage	26	86.8	189.48	5.25 (3.59, 7.16)	0.002
		Early stage	4	83.5	18.13	37.04 (5.37, 91.05)	
		Undetermined	25	85.7	167.77	0.49 (0.00, 1.66)	
	**Follow-up time**							
		< 3 years	5	92.1	50.70	20.23 (0.00, 75.29)	0.083
		> 5 years	35	89.3	318.60	3.39 (2.08, 4.95)	
		3–5 years	15	85.3	95.29	5.71 (2.26, 10.35)	
	**Endoscopy interval**							
		≤ 1 year	21	86.8	151.31	9.58 (4.82, 15.63)	0.002
		> 3 years	10	72.6	32.87	2.86 (1.16, 5.11)	
		2–3 years	13	90.0	120.37	2.59 (1.22, 4.38)	
		NR	11	91.5	118.33	0.82 (0.00, 3.68)	
	**Study quality**							
		high	19	96.5	515.93	2.24 (0.85, 4.10)	0.001
		moderate	36	87.9	289.52	5.52 (3.04, 8.53)	

*Test for subgroup differences (random effects model)

K, number of study; NR, not reported; IM, intestinal metaplasia; GC, gastric cancer.

The progressed rate to dysplasia in IM patients was estimated to be 12.51 (95% CI, 5.45, 22.03; I^2^: 95.1%) cases per 1,000 person-years in the pooled of 20 studies. The pooled results of the 10 studies using random-effect model showed that the regressed proportion was 31.83 (95% CI, 25.48–38.51%; I^2^: 91.0) and the persistence proportion was 43.46 (95% CI, 32.52–54.71%; I^2^: 96.0%) per 100 observations in the pooled 20 studies among patients with IM (**S1 Table and Fig 2**).

### Meta-regression analysis

To assess the effect of the potential covariates such as the number of participant in study and the year of study as a source of heterogeneity across included studies were used meta-regression analyses. For Incidence rate of GC in GA patients, the findings of meta-regression showed that the number of participant in study (p = 0.01; R^2^ = 0.31%) and the year of study (p = 0.001; R^2^ = 16.63%) partly demonstrated the heterogeneity, but for the incidence rate of GC in IM patients only the year of study had significant effect (p = 0.01; R^2^ = 14.31%).

### Sensitivity and subgroup analyses

After applying subgroup analyses by potential moderator variables, heterogeneity decreased among some of the strata of subgroups. The detailed findings of subgroups analyses are shown in **Tables 2 and 3.**

For GA patients, when stratified by region, the largest incidence rate of GC was 2.25 (95%CI; 1.67, 2.90) per 1,000 person-years in Asia studies compared with other strata. Similar to GA findings, the largest incidence rate of GC in IM studies was 7.58 (95%CI; 4.10, 11.91) per 1,000 person-years in Asia studies. In stratified analyses by the study design, highest incidence rate of GC in GA studies was observed in the retrospective cohorts as 2.72 (95%CI; 1.79, 3.81) per 1,000 person-years versus other strata. When stratified by IM studies, the largest incidence rate of GC in prospective cohorts was 4.62 (95 CI%; 2.01, 7.99) per 1,000 person-years compared with other groups. When stratified by type of GA and IM lesions, the highest incidence rate of GC was observed in severe GA (4.82 per 1,000 person-years) and IM incomplete patients (6.60 cases per 1,000 person-years) compared to other strata. When stratified by male percent among study participants, for GA patients, the largest incidence rate of GC was estimated as 2.84 (95%CI; 1.35, 4.70) per 1,000 person-years in non-reported strata, for IM studies, the highest incidence rate of GC was estimated as 282.17 (95%CI; 0.00, 1135.86) cases per 1,000 person-years in <40% male strata, this difference may, therefore, be the limited number of included studies across IM in our meta-analysis.

When stratified by age groups, in both GA and IM studies, the lowest incidence rate of GC was observed as 0.94 (95 CI%; 0.45, 1.56) and 2.30 (95 CI%; 1.08, 3.85) cases per 1,000 person-years in the >50 years strata compared with other strata, respectively. Also, when stratified by the stage of GC, in both GA and IM studies, the lowest incidence rate of GC was observed as 0.00 (95 CI%; 0.00, 1.10) and 0.49 (95 CI%; 0.00, 1.66) cases per 1,000 person-years in the undetermined strata compared with other strata, respectively.

In stratified analyses by follow-up time, highest incidence rate of GC in GA studies was observed in the >5 years follow-up of 1.63 (95 CI%; 1.02, 2.33) per 1,000 person-years versus other strata. When stratified by IM studies, the largest incidence rate of GC was in prospective cohorts <3 years follow-up of 20.23 (95 CI%; 0.00, 75.29) per 1000 person-years compared with other groups.

For GA patients, when stratified by endoscopy interval, the largest incidence rate of GC was 1.89 (95 CI%; 1.23, 2.65) per 1,000 person-years in ≤1 year interval strata compared with other strata. Similar to GA findings, the largest incidence rate of GC in IM studies was 9.58 (95 CI%; 4.82, 15.63) per 1,000 person-years in ≤1 year interval strata.

Subgroup analysis of the progress rate, regress and persistence proportion in both GA and IM patients is presented in **S1 Table**.

In sensitivity analysis, to examine the impact of each study on the strength of the pooled studies, related indicators were estimated after excluding each study from the analysis. Excluding some of the studies in GA or IM patients had significant influence on the pooled related summary effect size. We found that in the pooled incidence rate of GC in GA patients, there was a significant difference between the pre-sensitivity pooled incidence rate of GC in GA patients (1.24; 95% CI, 0.80, 1.76) and post-sensitivity pooled incidence rate of GC in GA patients of 39.56`; 95% CI, 17.27, 70.31) per 1,000 person-years after omitting Cheli et al. [80] study. The progressed rate in GA patients to dysplasia showed similar findings between the pre- (6.23; 95% CI, 2.34, 11.46) and post-sensitivity analysis (200.0; 95% CI, 42.17, 455.21) per 1,000 person-years after omitting Leung (b) et al. [26]. In the pooled incidence rate of GC in IM patients, there was a significant difference between the pre- (3.38; 95% CI, 2.13, 4.85) and post-sensitivity analysis (666.6`; 95% CI, 220.3, 1327.6) per 1,000 person-years after omitting Filipe(b) et al. [34] study. However, the lower and higher pooled related indications are shown in **Table 4**.

**Table 4:** The estimation of related indictors in patients with GA and IM based on sensitivity analysis.

Variables	Pre-sensitivity analysis			Upper & lower of effect size	Post-sensitivity analysis		
	k	Pooled ES (random effect)	95% CI		Pooled ES (random effect)	95% CI	Excluded studies
Incidence rate GC in GA patients*	90	1.24	0.80, 1.76	Upper	39.56`	17.27, 70.31	Cheli et al. [80]
				Lower	0.00	0.0, 0.41	Mera_(a)_ et al. [29]
Progress rate in GA patients to IM*	9	41.42	23.11, 64.45	Upper	47.30	27.56, 71.78	Gonzalez_(a)_ et al. [22]
				Lower	37.22	18.99, 60.73	You_(b)_ et al. [43]
Progress rate in GA patients to Dys*	11	6.23	2.34, 11.46	Upper	200.0	42.17, 455.21	Leung_(b)_ et al. [26]
				Lower	11.75	7.30, 17.23	Gonzalez_(a)_ et al. [22]
Regress proportion in GA patients**	10	32.23	18.07, 48.02	Upper	37.71	27.60, 48.31	Correa_(b)_ et al. [27]
				Lower	27.46	14.10, 42.93	Kokkola_(b)_ 2002 [60]
Persistence proportion in GA patients**	10	38.83	20.20, 59.13	Upper	42.44	23.10, 62.98	Leung_(b)_ et al. [26]
				Lower	32.89	14.63, 53.92	Valle et al. [84]
Incidence rate GC in IM patients*	55	3.38	2.13, 4.85	Upper	666.6	220.3, 1327.6	Filipe_(b)_ et al. [34]
				Lower	0.00	0.00, 1.65	Dinis-Ribeiro_(b)_ et al. [36]
Progress rate in IM patients to Dys	20	12.51	5.45, 22.03	Upper	13.92	6.39, 23.92	Gonzalez_(b)_ et al. [52]
				Lower	10.18	4.82, 17.17	You_(d)_ et al. [43]
Regress proportion in IM patients**	21	31.83	25.48, 38.51	Upper	32.51	26.06, 39.30	Dinis-Ribeiro_(b)_ et al. [36]
				Lower	28.42	24.05,32.99	Dinis-Ribeiro _(b)_ et al. [36]
Persistence proportion in IM patients**	20	43.46	32.52, 54.71	Upper	45.86	36.13; 55.75	You_(b)_ et al. [43]
				Lower	40.04	30.07, 50.44	Pittayanon_(a)_ et al. [37]

*rate per 1000 person-years

**proportion per 100 population

K, number of study; ES, effect size; GA, gastric atrophy; IM, intestinal metaplasia; Dys, dysplasia

### Publication bias

Begg's and Egger's statistics showed that there were no significant evidence of publication bias for assessing the incidence rate of GC in GA patients (Begg's: P = 0.88 and Egger's: P = 0.19), on progress rate in GA patients to IM (Begg's: P = 0.98 and Egger's: P = 0.56), on progress rate in GA patients to dysplasia (P = 0.39 and Egger's: P = 0.83), on regress proportion in GA patients (Begg's: P = 0.85 and Egger's: P = 0.70), on persistence proportion in GA patients (Begg's: P = 0.85 and Egger's: P = 0.27), on progress rate in IM patients to dysplasia (Begg's: P = 0.119 and Egger's: P = 0.587) on regress proportion in IM patients (Begg's: P = 0.52 and Egger's: P = 0.49), on persistence proportion in IM patients (Begg's: P = 0.47 and Egger's: P = 0.56) in our meta-analyses.

Significant publication bias was observed in incidence rate of GC in IM patients (Begg's: P = 0.002 and Egger's: P = 0.039), fourteen additional censored studies were used to conduct the trim and fill method; however, this did not change our robust pooled effect size. However, **v**isual inspection of the funnel plots is shown in **S1 Fig**.

## Discussion

Screening for GC in high risk groups especially those with premalignant lesions in the index endoscopy has been of interest for years as the late diagnosis of GC is associated with high mortality [89].

In this report in a total number of 87326 individuals with IM, we analyzed the rate of progression to GC which occurred in 1325 during a follow-up time from 6 months to 16.8 years (a total of 358317.55 person-years). This indicates that the incidence rate of GC in IM patients was 3.38 (95% CI, 2.13, 4.85; I2: 93.4%) cases per 1,000 person-years. Further progression rate to dysplasia was 12.51 (95% CI, 5.45, 22.03; I2: 95.1%) cases per 1,000 person-years in the pooled of 20 studies of IM patients. Summarizing the data of fifty articles involving 90 studies and a total number of 68893 patients, 920 cases of GC were found in patients with GA over the follow-up period ranging from 1 year to 21 years (a total of 454679.2 person-years). This corresponds to pooled GC incidence rate in patients with GA of 1.24 (95% CI, 0.80, 1.76; I2: 83.6%) cases per 1,000 person-years with random-effects model. It was not clear how many of these patients passed through the stage of IM, but even in patients with GA alone, there should be a screening strategy. Given that commencing a cascade toward GA is very low in the absence of *H*. *pylori* infection, adjustment for *H*. *pylori* infection seems essential for determining the risk of GC incidence in patients with GA [90, 91].

Over the last two decades, there has been a steady decline in the global incidence of GC. The widespread use of antibiotics in both developed and developing countries and improved control of *H*. *pylori* infection have strongly contributed to this decrease. Even if this trend continues, lack of specific screening programs for GC across geographical regions with low incidence is a crucial challenge [92].

Compared with other cancers, GC is associated with marked differences across different geographic regions. These statistics are also consistent with our analysis which revealed remarkable discrepancies across geographical regions [93].

The studies from Asia had highest rate of progression of IM to GC at 7.58 (95%CI; 4.10, 11.91) per 1,000 person-years. This was also true for progression of GA to IM, when compared to studies from Europe [94].

Establishment of screening programs differs based on regional incidence of GC which is particularly evident from Asian countries such as Korea and Japan where mass screening is widely available and cost-effective due to higher incidence. In contrast, late detection occurs frequently in western countries which results in poor patient survival as early stages of GC typically present with minimal or no symptoms [95, 96].

Our data demonstrated significant association between male gender and progression of IM to GC.

The enigmatic male dominance in incidence of GC cannot be entirely attributed to the differences in the gender-associated risk factors. Hormonal influence has been considered as a major role player [97]. Estrogen receptors, Era and ERb, are present in gastric tissue. Estrogen may exert its effect by protecting mucous epithelia via an increased expression of trefoil factor proteins. Furthermore, *H*. *pylori* infection is a well-known factor in promoting male-predominant gastric adenocarcinoma in humans. It has been shown that prevention of *H*. *pylori*-induced GC may be mediated by estrogen signaling that can diminish gastric levels of CXCL1, a neutrophil chemokine, leading to a decline in neutrophil infiltration and downregulation of oncogenic pathways [98, 99].

Camargo et al conducted a meta-analysis investigating the associations of use of estrogen- and antiestrogen-related therapies and also with menstrual and reproductive factors with gastric cancer in women. This study supported the hypothesis of decreased risk of GC after exposure to either ovarian or exogenous estrogen. Both hormone replacement therapy and longer years of fertility significantly reduced the risk of GC. On the other hand, tamoxifen treatment was associated with increased risk [100].

In line with other studies, the presence of incomplete metaplasia indicated a higher risk of progression of IM to GC [91].

A 2013 study by O’Connor et al indicated that in spite of a definite risk of progression from IM to cancer, there is a lack of high quality data on the utility of endoscopic or biochemical surveillance for detection of dysplasia and early gastric cancer to decrease mortality in patients with gastric IM. Based on a review carried out on patients in the US, The American Society for Gastrointestinal Endoscopy (ASGE) recommends surveillance of IM with histological evaluation in those with a family history or ethnic predisposition to gastric cancer or with low grade dysplasia without suggesting an appropriate surveillance interval [101].

The usefulness of subtyping of IM has been reviewed in 2013 by González et al who assessed the risk of GC among subjects with different types of IM. According to this comprehensive review, the relative risks of GC were significantly higher for the presence of incomplete type in comparison to complete type or the absence of incomplete type which shows scientific evidence supporting the utility of subtyping IM as a predictor of GC risk. This is contrary to recently published guidelines for endoscopic management of precancerous gastric lesions that do not suggest subtyping of IM. However, these recommendations are mainly based on retrospective studies which show inconsistent results [102].

Older patients are more likely to be diagnosed with GC compared to their younger counterparts and the majority of affected patients belong to this age group. Moreover, age is also identified as an independent prognostic factor for distant metastasis in GC. Despite limited data, several reports indicated that older individuals benefit from different therapeutic options in the same way as their younger counterparts and hence chronological age should not be a limiting factor to withhold curative or palliative treatment of GC [103, 104].

Interestingly, there was lower rate of progression to malignancy in those aged more than fifty years and in the first three years entering follow up endoscopies. This may be related to ascertainment bias in the cohort of referral patients. It could be that in referral cohorts, patients might have already become symptomatic because of GC even in the earliest stage which was not diagnosed at the first endoscopy. This needs to be clarified in prospective large cohorts.

There was possibility of regression in both GA and IM in our series. For GA, the pooled results of the 10 studies using random-effect model revealed an estimated regressed proportion of 32.23 (95% CI, 18.07–48.02; I2: 94.0%) with a persistence proportion of 38 from 83 (95% CI, 20.20–59.13; I2: 97.0%) per 100 observations. Using random-effect model in the pooled 20 studies among patients with IM, it was revealed that 31.83 (95% CI, 25.48–38.51%; I2: 91.0) of patients with IM regressed while 43.46 (95% CI, 32.52–54.71%; I^2^: 96.0%) had persistent IM per 100 observations.

Some studies reported that the precancerous lesions including GA and IM had improved after eradication of *H*.*pylori*, but other studies did not find any change [105]. Discrepancies such as completeness of eradication, stage of the disease when treatment was initiated, and the short follow-up period that did not exceed 1 year are among reasons that have hampered attempts to reach a consensus about the improvement of GA or IM after eradication [106].

A 2009 meta-analysis by Fuccio et al that included studies in areas with high incidence of gastric cancer, mostly in Asia evaluated the effect of *H*.*pylori* eradication treatment during follow-up and showed reduced risk of GC progression and increased probability of regression in preneoplastic lesions [107].

In contrast to some previous reports indicating that IM could not regress, the reversibility of IM has been shown in recent studies both in human and animal [13, 108]. There are two meta-analyses by Rokkas et al (2007)[109] and Wang et al (2011)[110] regarding the long-term effects of *H*. *pylori* eradication on gastric histology that showed significant improvement of GA but without considerable histological alterations in IM after *H*. *pylori* eradication. However, Watari et al reported that when those studies with follow-up of more than 5 years following *H*. *pylori* eradication were considered, both GA and IM tended to improve histologically [106]. Further studies characterizing those who have regressed may be of utmost importance in preventing the malignant transformation cascade.

With regard to the importance of screening, a 2018 meta-analysis by Zhang et al including 10 cohort or case-control studies from Asian countries evaluated the relationship between endoscopic screening for gastric cancer and mortality incidence. Results indicated that relative risk reduction in gastric cancer mortality after endoscopic screening was 40%. Also, endoscopic screening resulted in significant decrease of gastric cancer mortality in comparison to no screening or radiographic screening [96].

The risk for GC is strikingly higher in patients with incomplete-type IM, those with involvement of antral and gastric body, first-degree relative of gastric cancer patients, and extension of IM over 20% of gastric mucosa. Consequently, annual endoscopic control would appear justified in such patients and in the remaining patients, a less intensive for instance every 2–3 years is proposed [111].

Interestingly, most cases of GC were detected in the first 3 years after diagnosis of IM or GA in our analysis. As discussed earlier, this might be related to the fact that first endoscopies were done usually in symptomatic patients rather than totally asymptomatic population. This may lead to length and lead time biases in these studies.

However, considering the difference in frequency of various types of precancerous lesions, justifying a surveillance strategy needs further large prospective, randomized, multicenter investigations based on lesion characteristics to better explore a screening protocol for those lesions that may predispose patients to malignant progression [111].

To the best of our knowledge, this is the most extensive review on this topic. The most recent published systematic review on this issue used only 15 studies comprising 19,749 participants, but we included 68 articles including 101317 participants in the current meta-analysis [91].

There are several limitations to our study. This meta-analysis has not been registered online. There was no distinction between multifocal IM and limited IM in most of these studies. Furthermore, there was no distinction between types of IM in these studies. Several of these studies relied on gastric mapping with standard endoscopy but did not use the Gastric Intestinal Metaplasia Assessment (OLGIM) classification of premalignant gastric lesions which was recommended by international societies [18].

Furthermore, there are scant reports of use of chromoendoscopy or advanced endoscopy in detection of premalignant lesions or their progression. Use of these techniques may increase the yield of detection or alternatively may reduce the need for multiple biopsies or increase the intervals for subsequent endoscopies.

## Conclusion

Although the overall incidence of GC after diagnosis of IM and GA is low, screening for this lethal cancer has gathered considerable momentum. The association varies between continents and is more frequent in Asian males and elderly. More intensified screening in these groups especially in the first three years after diagnosis along with eradication of *H*.*pylori* may reduce the burden of this deadly disease.

## Supporting information

S1 Table The estimation of progress rate among patients with GA to IM and dysplasia and IM to dysplasia based on subgroup analyses. (DOC)

S2 Table Search strategies. (DOCX)

S3 Table PRISMA 2009 checklist. (DOCX)

S1 Fig Funnel plot for assessing the publication bias in meta-analysis on incidence rate GC in GA patients (A) and in IM patients (B).
(TIF)

S1 Dataset Datasets providing the information of all the studies included in the meta-analyses for studying outcomes. (RAR)
