# Supplementary material for: Gastric cancer in patients with gastric atrophy and intestinal metaplasia: A systematic review and meta-analysis
Source: PLoS One. 2019 Jul 26;14(7):e0219865. doi: 10.1371/journal.pone.0219865 (PMC6660080; doi:10.1371/journal.pone.0219865)
Supplement: S1 Table — (DOC) [file pone.0219865.s001.doc]

**S1 Table**. The estimation of progress rate among patients with GA to IM and dysplasia and IM to dysplasia based on subgroup analyses

| **Variables** | | | **K** | **I^2^ (%)** | **Q test** | **Inc rate per 1000 or proportion per 100 observetions**  **(95% CI)** | **Test for subgroup differences**  **p-value** |  |
| --- | --- | --- | --- | --- | --- | --- | --- | --- |
| **Progress rate GA to IM*** | | |  |  |  |  |  |  |
|  | **Total** | | 9 | 95.6 | 180.03 | 41.42 (23.11, 64.45) | - |  |
|  | **Region** | |  |  |  |  |  |  |
|  |  | Asia | 3 | 81.9 | 11.03 | 58.52 (33.92, 88.92) | <0.0001 |  |
|  |  | Europe | 5 | 78.2 | 18.33 | 20.68 (11.15, 32.73) |  |  |
|  |  | Other | 1 | - | 0.00 | 68.85 (55.47, 83.68) |  |  |
|  | **Study design** | |  |  |  |  |  |  |
|  |  | Prospective cohort | 6 | 95.5 | 110.51 | 50.47 (28.33, 78.61) | 0.001 |  |
|  |  | Retrospective cohort | 2 | 80.9 | 5.25 | 17.10 (6.53, 32.46) |  |  |
|  |  | Prospective RCT | 1 | - | 0.00 | 200.0 (42.17, 455.21) |  |  |
|  | **Type GA** | |  |  |  |  |  |  |
|  |  | Undetermined | 6 | 95.0 | 99.81 | 34.71 (13.36, 64.37) | 0.058 |  |
|  |  | GA mild | 1 | - | 0.00 | 50.61 (44.33, 57.29) |  |  |
|  |  | GA moderate | 1 | - | 0.00 | 43.47 (16.19, 82.75) |  |  |
|  |  | GA severe | 1 | - | 0.00 | 72.64 (56.34, 91.00) |  |  |
|  | **Male (% study population)** | |  |  |  |  |  |  |
|  |  | < 40 % | - | - | - | - | 0.001 |  |
|  |  | 40-50 % | 4 | 77.9 | 13.55 | 17.45 (8.95, 28.35) |  |  |
|  |  | ≥ 50 % | 2 | 84.4 | 6.40 | 59.96 (40.21, 83.60) |  |  |
|  |  | NR | 3 | 59.7 | 4.96 | 61.42 (28.44, 105.10) |  |  |
|  | **Age (mean, years)** | |  |  |  |  |  |  |
|  |  | ≤ 50 | 5 | 97.1 | 136.02 | 34.62 (15.22, 61.62) | 0.082 |  |
|  |  | > 50 | 3 | 84.0 | 12.48 | 75.89 (4.59, 212.12) |  |  |
|  |  | NR | 1 | - | 0.00 | 68.85 (55.47, 83.68) |  |  |
|  | **Follow-up time** | |  |  |  |  |  |  |
|  |  | < 3 years | - | - | - | - | 0.028 |  |
|  |  | > 5 years | 5 | 95.5 | 88.04 | 29.07 (11.93, 53.37) |  |  |
|  |  | 3-5 years | 4 | 76.5 | 12.77 | 62.18 (37.79, 91.84) |  |  |
|  | **Endoscopy** interval | |  |  |  |  |  |  |
|  |  | ≤ 1 year | 1 | - | 0.00 | 16.46 (12.07, 21.53) | 0.0002 |  |
|  |  | > 3 years | 3 | 97.5 | 80.63 | 51.30 (4.20, 138.1) |  |  |
|  |  | 2-3 years | 4 | 86.5 | 22.22 | 49.75 (30.14, 73.95) |  |  |
|  |  | NR | 1 | - | 0.00 | 43.47 (16.19, 82.74) |  |  |
|  | **Study quality** | |  |  |  |  |  |  |
|  |  | high | 4 | 77.9 | 13.55 | 17.45 (8.95, 28.35) | <0.0001 |  |
|  |  | moderate | 5 | 73.5 | 15.12 | 57.51 (42.55, 74.52) |  |  |
| **Progress rate** **GA to Dys*** | | |  |  |  |  |  |  |
|  | **Total** |  | 11 | 83.0 | 58.71 | 6.23 (2.34, 11.46) |  |  |
|  | **Region** | |  |  |  |  |  |  |
|  |  | Asia | 3 | 8.3 | 2.18 | 7.09 (3.98, 10.82) | <0.0001 |  |
|  |  | Europe | 7 | 33.2 | 8.98 | 8.30 (5.27, 11.90) |  |  |
|  |  | Other | 1 | - | 0.00 | 0.00 (0.00, 1.29) |  |  |
|  | **Study design** | |  |  |  |  |  |  |
|  |  | Prospective cohort | 9 | 86.4 | 58.69 | 7.87 (3.38, 13.87) | 0.977 |  |
|  |  | Retrospective cohort | 1 | - | 0.00 | 9.65 (3.59, 18.37) |  |  |
|  |  | Prospective RCT | 1 | - | 0.00 | 0.00. (0.00, 85.94) |  |  |
|  | **Type GA** | |  |  |  |  |  |  |
|  |  | Undetermined | 8 | 83.1 | 41.44 | 3.55 (0.16, 9.69) | 0.132 |  |
|  |  | GA mild | 1 | - | 0.00 | 12.91 (9.84, 16.40) |  |  |
|  |  | GA moderate | 1 | - | 0.00 | 12.42 (0.18, 37.44) |  |  |
|  |  | GA severe | 1 | - | 0.00 | 19.23 (11.25, 29.27) |  |  |
|  | **Male (% study population)** | |  |  |  |  |  |  |
|  |  | < 40 % | 2 | 63.9 | 2.77 | 7.04 (2.68, 13.33) | 0.280 |  |
|  |  | 40-50 % | 2 | 0.00 | 0.20 | 5.40 (0.58, 13.32) |  |  |
|  |  | ≥ 50 % | 2 | 54.1 | 2.18 | 14.89 (9.42, 21.58) |  |  |
|  |  | NR | 5 | 89.7 | 38.70 | 3.62 (0.00, 17.17) |  |  |
|  | **Age (mean, years)** | |  |  |  |  |  |  |
|  |  | ≤ 50 | 3 | 8.9 | 2.20 | 13.17 (9.75, 17.10) | <0.0001 |  |
|  |  | > 50 | 7 | 30.6 | 8.64 | 5.56 (2.81, 8.98) |  |  |
|  |  | NR | 1 | - | 0.00 | 0.00 (0.00, 1.29) |  |  |
|  | **Follow-up time** | |  |  |  |  |  |  |
|  |  | < 3 years | - | - | - | - | 0.180 |  |
|  |  | > 5 years | 6 | 87.7 | 40.52 | 6.10 (1.73, 14.94) |  |  |
|  |  | 3-5 years | 5 | 0.00 | 3.72 | 7.10 (4.75, 9.77) |  |  |
|  | **Endoscopy** interval | |  |  |  |  |  |  |
|  |  | ≤ 1 year | 2 | 0.0 | 0.03 | 13.58 (9.20, 18.78) | 0.079 |  |
|  |  | > 3 years | 3 | 79.1 | 9.55 | 0.00 (0.00, 2.93) |  |  |
|  |  | 2-3 years | 5 | 4.8 | 4.20 | 10.78 (8.22, 13.64) |  |  |
|  |  | NR | 1 | - | 0.00 | 12.42 (0.18, 37.44) |  |  |
|  | **Study quality** | |  |  |  |  |  |  |
|  |  | high | 3 | 0.0 | 0.21 | 6.98 (3.27, 11.70) | 0.878 |  |
|  |  | moderate | 8 | 88.0 | 58.47 | 6.22 (1.44, 13.29) |  |  |
| **Progress rate IM to Dys*** | | |  |  |  |  |  |  |
|  | **Total** |  | 20 | 95.1 | 389.18 | 12.51 (5.45, 22.03) |  |  |
|  | **Region** | |  |  |  |  |  |  |
|  |  | Asia | 8 | 93.6 | 109.30 | 21.36 (6.10, 44.32) | <0.0001 |  |
|  |  | Europe | 10 | 85.6 | 62.70 | 5.07 (1.69, 9.90) |  |  |
|  |  | Other | 2 | 0.0 | 0.52 | 31.05 (22.93, 40.32) |  |  |
|  | **Study design** | |  |  |  |  |  |  |
|  |  | Prospective cohort | 11 | 96.5 | 284.63 | 15.72 (4.10, 33.85) | 0.014 |  |
|  |  | Retrospective cohort | 8 | 80.7 | 36.27 | 8.01 (2.88, 15.05) |  |  |
|  |  | Prospective RCT | 1 | - | 0.00 | 2.06 (0.03, 6.21) |  |  |
|  | **Type IM** | |  |  |  |  |  |  |
|  |  | IM | 5 | 93.2 | 58.79 | 10.73 (0.88, 29.14) | 0.701 |  |
|  |  | IM complete | 6 | 93.2 | 73.58 | 9.51 (1.15, 24.19) |  |  |
|  |  | IM incomplete | 9 | 96.4 | 219.75 | 16.57 (3.50, 37.42) |  |  |
|  | **Male (% study population)** | |  |  |  |  |  |  |
|  |  | < 40 % | - | - | - | - | 0.005 |  |
|  |  | 40-50 % | 5 | 82.8 | 23.21 | 12.27 (3.70, 25.05) |  |  |
|  |  | ≥ 50 % | 14 | 96.0 | 324.54 | 11.04 (2.86, 23.41) |  |  |
|  |  | NR | 1 | - | 0.00 | 33.55 (24.93, 43.45) |  |  |
|  | **Age (mean, years)** | |  |  |  |  |  |  |
|  |  | ≤ 50 | 5 | 77.6 | 17.88 | 33.68 (19.32, 51.73) | <0.0001 |  |
|  |  | > 50 | 14 | 82.3 | 73.63 | 4.74 (1.66, 8.98) |  |  |
|  |  | NR | 1 | - | 0.00 | 33.55 (24.93, 43.44) |  |  |
|  | **Follow-up time** | |  |  |  |  |  |  |
|  |  | < 3 years | 1 | - | 0.00 | 8.60 (0.12, 25.94) | 0.757 |  |
|  |  | > 5 years | 11 | 93.6 | 155.72 | 10.76 (3.82, 20.68) |  |  |
|  |  | 3-5 years | 8 | 95.4 | 150.78 | 15.47 (2.66, 36.55) |  |  |
|  | **Endoscopy** interval | |  |  |  |  |  |  |
|  |  | ≤ 1 year | 7 | 82.1 | 33.55 | 4.46 (0.00, 14.69) | 0.331 |  |
|  |  | > 3 years | 7 | 93.5 | 92.29 | 13.19 (3.96, 26.99) |  |  |
|  |  | 2-3 years | 6 | 96.1 | 129.19 | 19.69 (5.59, 41.79) |  |  |
|  |  | NR | - | - | - | - |  |  |
|  | **Study quality** | |  |  |  |  |  |  |
|  |  | high | 12 | 86.7 | 82.44 | 7.15 (2.87, 13.01) | 0.056 |  |
|  |  | moderate | 8 | 93.6 | 109.07 | 20.06 (6.97, 38.86) |  |  |
| **Regress proportion GA**** | | |  |  |  |  |  |  |
|  | **Total** |  | 10 | 94.0 | 142.35 | 32.23 (18.07, 48.02) | - |  |
|  | **Country/region** | |  |  |  |  |  |  |
|  |  | Asia | 2 | 81.2 | 5.31 | 25.20 (0.00, 81.46) | <0.0001 |  |
|  |  | Europe | 7 | 66.7 | 18.02 | 37.20 (26.35, 48.65) |  |  |
|  |  | Other | 1 | - | 0.00 | 8.77 (5.62, 12.53) |  |  |
|  | **Study design** | |  |  |  |  |  |  |
|  |  | Prospective cohort | 7 | 95.4 | 130.21 | 38.61 (19.88, 59.09) | 0.203 |  |
|  |  | Retrospective cohort | 2 | 83.3 | 5.99 | 24.30 (1.92, 58.13) |  |  |
|  |  | Prospective RCT | 1 | - | 0.00 | 0.00 (0.00, 38.85) |  |  |
|  | **Type metaplasia** | |  |  |  |  |  |  |
|  |  | GA | 6 | 93.6 | 77.75 | 20.60 (6.85, 38.29) | 0.089 |  |
|  |  | GA mild | - | - | - | - |  |  |
|  |  | GA moderate | 2 | 0.0 | 0.81 | 36.83 (18.97, 56.53) |  |  |
|  |  | GA severe | 2 | 82.6 | 5.74 | 66.84 (27.29, 96.65) |  |  |
|  | **Male (% study population)** | |  |  |  |  |  |  |
|  |  | < 40 % | - | - | - | - | 0.018 |  |
|  |  | 40-50 % | 3 | 0.00 | 1.01 | 35.77 (30.93, 40.76) |  |  |
|  |  | ≥ 50 % | 4 | 82.3 | 16.94 | 46.36 (20.88, 72.77) |  |  |
|  |  | NR | 3 | 53.5 | 4.30 | 9.81 (0.19, 26.74) |  |  |
|  | **Age (mean, years)** | |  |  |  |  |  |  |
|  |  | ≤ 50 | 4 | 74.0 | 11.56 | 39.66 (31.12, 48.53) | <0.0001 |  |
|  |  | > 50 | 5 | 79.8 | 19.84 | 34.29 (6.70, 68.10) |  |  |
|  |  | NR | 1 | - | 0.00 | 8.78 (5.62, 12.54) |  |  |
|  | **follow-up time** | |  |  |  |  |  |  |
|  |  | < 3 years | 2 | 75.6 | 4.10 | 68.09 (23.87, 99.24) | 0.153 |  |
|  |  | > 5 years | 5 | 94.7 | 75.65 | 22.87 (8.70, 40.85) |  |  |
|  |  | 3-5 years | 3 | 65.7 | 5.84 | 33.99 (10.54, 61.63) |  |  |
|  | **Endoscopy** interval | |  |  |  |  |  |  |
|  |  | ≤ 1 year | 1 | - | 0.00 | 34.08 (27.99, 40.44) | 0.002 |  |
|  |  | > 3 years | 4 | 94.6 | 55.92 | 13.24 (0.00, 38.23) |  |  |
|  |  | 2-3 years | 2 | 0.00 | 0.65 | 48.83 (41.94, 55.73) |  |  |
|  |  | NR | 3 | 76.2 | 8.39 | 54.19 (20.19, 86.37) |  |  |
|  | **Study Quality** | |  |  |  |  |  |  |
|  |  | high | 3 | 0.0 | 1.01 | 35.77 (30.93, 40.76) | 0.712 |  |
|  |  | moderate | 7 | 95.3 | 128.70 | 30.16 (8.50, 56.94) |  |  |
| **Regress proportion IM**** | | |  |  |  |  |  |  |
|  | **Total** |  | 21 | 91.0 | 210.94 | 31.83 (25.48, 38.51) |  |  |
|  | **region** |  |  |  |  |  |  |  |
|  |  | Asia | 7 | 84.3 | 38.18 | 28.81 (20.04, 38.39) | 0.026 |  |
|  |  | Europe | 11 | 92.6 | 135.13 | 37.47 (26.80, 48.77) |  |  |
|  |  | Other | 3 | 89.7 | 38.70 | 18.21 (10.57, 25.79) |  |  |
|  | **Study design** | |  |  |  |  |  |  |
|  |  | Prospective cohort | 11 | 57.8 | 23.69 | 30.75 (26.86, 34.80) | <0.0001 |  |
|  |  | Retrospective cohort | 9 | 94.3 | 141.52 | 33.64 (17.71, 51.59) |  |  |
|  |  | Prospective RCT | 1 | - | 0.00 | 13.40 (8.93, 18.60) |  |  |
|  | **Type IM** | |  |  |  |  |  |  |
|  |  | Undetermined | 6 | 85.6 | 34.80 | 25.82 (17.79, 34.73) | 0.197 |  |
|  |  | IM complete | 6 | 58.5 | 12.05 | 27.42 (21.14, 34.15) |  |  |
|  |  | IM incomplete | 9 | 94.1 | 136.41 | 40.66 (26.25, 55.90) |  |  |
|  | **Male (% study population)** | |  |  |  |  |  |  |
|  |  | < 40 % | - | - | - | - | 0.106 |  |
|  |  | 40-50 % | 5 | 95.9 | 97.21 | 48.10 (19.91, 76.92) |  |  |
|  |  | ≥ 50 % | 14 | 81.1 | 68.72 | 27.80 (22.52, 33.39) |  |  |
|  |  | NR | 2 | 0.00 | 0.81 | 22.73 (18.21, 27.57) |  |  |
|  | **Age (mean, years)** | |  |  |  |  |  |  |
|  |  | ≤ 50 | 5 | 50.7 | 8.12 | 31.55 (25.42, 38.01) | 0.030 |  |
|  |  | > 50 | 14 | 93.3 | 192.60 | 33.58 (23.68, 44.21) |  |  |
|  |  | NR | 2 | 0.00 | 0.81 | 22.73 (18.21, 27.57) |  |  |
|  | **follow-up time** | |  |  |  |  |  |  |
|  |  | < 3 years | 1 | - | 0.00 | 26.73 (18.50, 35.83) | 0.257 |  |
|  |  | > 5 years | 11 | 92.8 | 139.08 | 36.99 (25.69, 49.01) |  |  |
|  |  | 3-5 years | 9 | 86.4 | 58.73 | 26.10 (18.78, 34.10) |  |  |
|  | **Endoscopy** interval | |  |  |  |  |  |  |
|  |  | ≤ 1 year | 8 | 71.5 | 24.52 | 27.38 (19.00, 36.59) | 0.253 |  |
|  |  | > 3 years | 7 | 87.3 | 47.21 | 27.45 (18.99, 36.76) |  |  |
|  |  | 2-3 years | 6 | 95.9 | 122.38 | 41.88 (26.27, 58.34) |  |  |
|  |  | NR | - | - | - | - |  |  |
|  | **Study Quality** | |  |  |  |  |  |  |
|  |  | high | 12 | 91.3 | 125.98 | 36.90 (26.93, 47.43) | 0.062 |  |
|  |  | moderate | 9 | 86.0 | 57.24 | 24.95 (17.96, 32.63) |  |  |
|  |  |  |  |  |  |  |  |  |
| **Persistence proportion in GA**** | | |  |  |  |  |  |  |
|  | **Total** |  | 10 | 97.0 | 359.30 | 38.83 (20.20, 59.13) | - |  |
|  | **Country/region** | |  |  |  |  |  |  |
|  |  | Asia | 3 | 99.3 | 301.85 | 22.82 (0.00, 78.38) | 0.441 |  |
|  |  | Europe | 7 | 80.0 | 29.97 | 45.14 (32.34, 58.28) |  |  |
|  |  | Other | - | - | - | - |  |  |
|  | **Study design** | |  |  |  |  |  |  |
|  |  | Prospective cohort | 6 | 98.5 | 324.07 | 32.75 (9.34, 61.42) | 0.048 |  |
|  |  | Retrospective cohort | 3 | 91.1 | 22.40 | 60.02 (32.87, 84.37) |  |  |
|  |  | Prospective RCT | 1 | - | 0.00 | 0.00 (0.00, 38.85) |  |  |
|  | **Type GA** | |  |  |  |  |  |  |
|  |  | Undetermined | 6 | 83.1 | 29.59 | 44.32 (29.78, 59.31) | <0.0001 |  |
|  |  | GA mild | 1 | - | 0.00 | 67.34 (64.45, 70.17) |  |  |
|  |  | GA moderate | 1 | - | 0.00 | 53.84 (26.10, 80.52) |  |  |
|  |  | GA severe | 2 | 0.0 | 0.25 | 7.38 (3.76, 11.80) |  |  |
|  | **Male (% study population)** | |  |  |  |  |  |  |
|  |  | < 40 % | - | - | - | - | 0.143 |  |
|  |  | 40-50 % | 4 | 69.8 | 9.94 | 41.30 (31.50, 51.43) |  |  |
|  |  | ≥ 50 % | 5 | 98.7 | 311.20 | 45.70 (9.96, 83.99) |  |  |
|  |  | NR | 1 | - | 0.00 | 0.00 (0.00, 38.85) |  |  |
|  | **Age (mean, years)** | |  |  |  |  |  |  |
|  |  | ≤ 50 | 4 | 99.1 | 330.87 | 37.20 (12.10, 66.75) | 0.828 |  |
|  |  | > 50 | 6 | 82.3 | 28.27 | 40.67 (15.70, 68.05) |  |  |
|  |  | NR | - | - | - | - |  |  |
|  | **follow-up time** | |  |  |  |  |  |  |
|  |  | < 3 years | 2 | 75.6 | 4.10 | 31.90 (0.75, 76.12) | 0.368 |  |
|  |  | > 5 years | 4 | 86.8 | 22.69 | 52.38 (37.47, 67.09) |  |  |
|  |  | 3-5 years | 4 | 99.0 | 308.38 | 21.69 (0.00, 69.68) |  |  |
|  | **Endoscopy** interval | |  |  |  |  |  |  |
|  |  | ≤ 1 year | 1 | - | 0.00 | 42.15 (35.73, 48.70) | 0.969 |  |
|  |  | > 3 years | 3 | 90.7 | 21.49 | 44.18 (3.05, 90.47) |  |  |
|  |  | 2-3 years | 4 | 99.0 | 301.55 | 36.66 (5.06, 76.46) |  |  |
|  |  | NR | 2 | 75.6 | 4.10 | 31.90 (0.75, 76.12) |  |  |
|  | **Study Quality** | |  |  |  |  |  |  |
|  |  | high | 4 | 69.8 | 9.94 | 41.29 (31.49, 51.42) | 0.882 |  |
|  |  | moderate | 6 | 98.4 | 318.13 | 37.82 (6.09, 75.97) |  |  |
| **Persistence proportion in IM**** | | |  |  |  |  |  |  |
|  | **Total** |  | 20 | 96.0 | 518.20 | 43.46 (32.52, 54.71) | - |  |
|  | **region** |  |  |  |  |  |  |  |
|  |  | Asia | 7 | 98.4 | 384.00 | 52.91 (27.04, 78.01) | <0.0001 |  |
|  |  | Europe | 11 | 86.9 | 76.57 | 45.67 (37.08, 54.40) |  |  |
|  |  | Other | 2 | 0.0 | 0.00 | 8.95 (2.89, 17.40) |  |  |
|  | **Study design** | |  |  |  |  |  |  |
|  |  | Prospective cohort | 10 | 96.0 | 223.08 | 43.22 (29.35, 57.64) | <0.0001 |  |
|  |  | Retrospective cohort | 9 | 95.7 | 186.78 | 38.62 (21.22, 57.60) |  |  |
|  |  | Prospective RCT | 1 | - | 0.00 | 81.44 (75.64, 86.62) |  |  |
|  | **Type MI** | |  |  |  |  |  |  |
|  |  | Undetermined | 5 | 90.4 | 41.76 | 61.55 (47.01, 75.15) | 0.016 |  |
|  |  | IM complete | 7 | 97.9 | 290.65 | 39.40 (15.01, 66.80) |  |  |
|  |  | IM incomplete | 8 | 78.2 | 32.05 | 37.38 (29.62, 45.45) |  |  |
|  | **Male (% study population)** | |  |  |  |  |  |  |
|  |  | < 40 % | - | - | - | - | 0.852 |  |
|  |  | 40-50 % | 5 | 91.4 | 46.50 | 38.06 (19.02, 59.08) |  |  |
|  |  | ≥ 50 % | 14 | 97.2 | 466.90 | 45.37 (31.61, 59.49) |  |  |
|  |  | NR | 1 | - | 0.00 | 42.31 (23.80, 61.93) |  |  |
|  | **Age (mean, years)** | |  |  |  |  |  |  |
|  |  | ≤ 50 | 5 | 96.5 | 113.59 | 34.57 (14.56, 57.74) | 0.663 |  |
|  |  | > 50 | 14 | 95.8 | 307.45 | 46.69 (33.89, 59.70) |  |  |
|  |  | NR | 1 | - | 0.00 | 42.31 (23.80, 61.93) |  |  |
|  | **follow-up time** | |  |  |  |  |  |  |
|  |  | < 3 years | 1 | 7- | 0.00 | 71.28 (62.03, 79.73) | <0.0001 |  |
|  |  | > 5 years | 10 | 51.1 | 18.41 | 41.64 (36.08, 47.29) |  |  |
|  |  | 3-5 years | 9 | 98.3 | 471.49 | 44.27 (23.92, 65.64) |  |  |
|  | **Endoscopy** interval | |  |  |  |  |  |  |
|  |  | ≤ 1 year | 8 | 96.4 | 194.99 | 43.58 (21.15, 67.43) | 0.469 |  |
|  |  | > 3 years | 6 | 93.6 | 78.70 | 51.66 (35.19, 67.92) |  |  |
|  |  | 2-3 years | 6 | 97.4 | 192.66 | 35.34 (17.39, 55.62) |  |  |
|  |  | NR | - | - | - | - |  |  |
|  | **Study Quality** | |  |  |  |  |  |  |
|  |  | high | 12 | 85.3 | 74.75 | 45.99 (37.73, 54.35) | 0.724 |  |
|  |  | moderate | 8 | 98.4 | 436.82 | 41.28 (18.47, 66.18) |  |  |

*rate per 1000 person-years

**proportion per 100 population

Test for subgroup differences (random effects model)

K, number of study; NR, not reported; ES, effect size; GA, gastric atrophy; IM, intestinal metaplasia; Dys, dysplasia.
