# Supplementary material for: Gastric cancer in patients with gastric atrophy and intestinal metaplasia: A systematic review and meta-analysis
Source: PLoS One. 2019 Jul 26;14(7):e0219865. doi: 10.1371/journal.pone.0219865 (PMC6660080; doi:10.1371/journal.pone.0219865)
Supplement: S2 Table — (DOCX) [file pone.0219865.s002.DOCX]

**S1 Table**. Search strategies.

| **Database** | **Search Strategy** |
| --- | --- |
| **PubMed** | ((((((((((((("Gastritis, Atrophic"[MeSH Terms]) OR "Gastritis, Atrophic"[Title/Abstract]) OR "Atrophic Gastrit*"[Title/Abstract]) OR "Atrophy"[MeSH Terms]) OR "Atroph*"[Title/Abstract]) OR "gastric atroph*"[Title/Abstract]) OR "Metaplasia"[MeSH Terms]) OR "Metaplasia"[Title/Abstract]) OR "Intestine"[MeSH Terms]) OR "Intestin*"[Title/Abstract]) OR "intestinal metaplasia"[Title/Abstract])) AND (((((("Stomach"[MeSH Terms]) OR "Stomach*"[Title/Abstract]) OR "gastri*") OR Esophagogastric Junction[Title/Abstract]) OR "Esophagogastric Junction"[MeSH Terms]) OR Esophagogastri*[Title/Abstract])) AND (((((((((((("Neoplasms"[MeSH Terms]) OR "Neoplasms"[Title/Abstract]) OR "cancer*") OR "Tumor*") OR "Neoplasia") OR "Malignan*"[Title/Abstract]) OR "carcinoma"[MeSH Terms]) OR "carcinoma*"[Title/Abstract]) OR "adenocarcinoma"[MeSH Terms]) OR "adenocarcinoma*"[Title/Abstract]) OR "Adenoma*"[Title/Abstract])) |
| **EMBASE** | #1 ('atrophic gastritis'/exp OR 'atrophy'/exp OR 'atrophy' OR 'metaplasia'/exp OR 'metaplasia' OR 'atrophic gastrit*':ab,ti OR 'atroph*':ab,ti OR 'gastric atroph*':ab,ti OR 'metaplasia':ab,ti OR 'intestine':ab,ti OR 'intestin*':ab,ti OR 'intestinal metaplasia':ab,ti)  #2 ('stomach*' OR 'gastri*' OR esophagogastric) NEAR/2 ('neoplasms' OR 'cancer*' OR 'tumor*' OR 'neoplasia' OR 'malignan*' OR 'carcinoma*' OR 'adenocarcinoma*' OR 'adenoma*')  #1 AND #2 |
