# Supplementary figures and images for: Gastric cancer in patients with gastric atrophy and intestinal metaplasia: A systematic review and meta-analysis
Source: PLoS One. 2019 Jul 26;14(7):e0219865. doi: 10.1371/journal.pone.0219865 (PMC6660080; doi:10.1371/journal.pone.0219865)

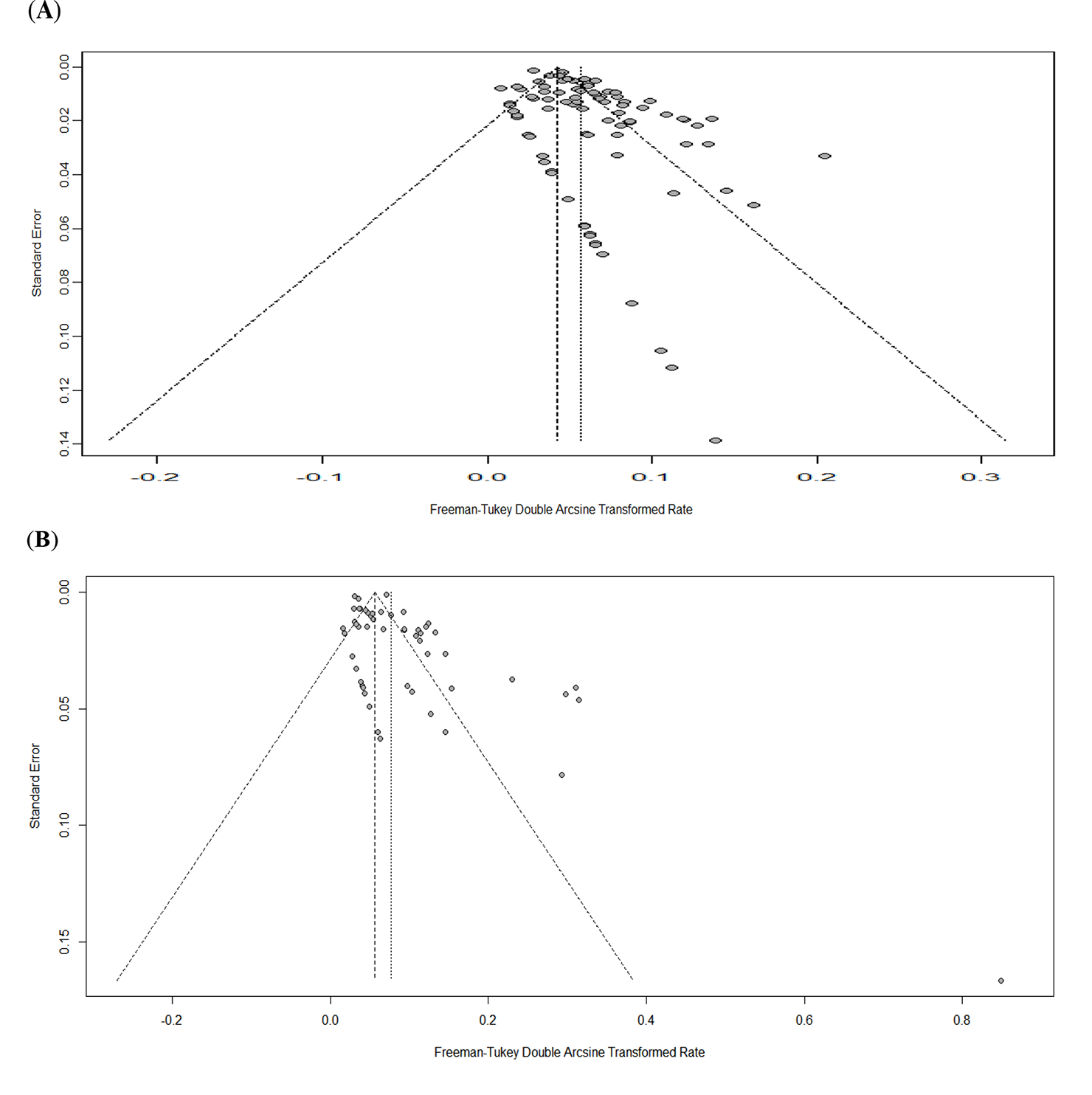

Supplement: S1 Fig — Funnel plot for assessing the publication bias in meta-analysis on incidence rate GC in GA patients (A) and in IM patients (B). (TIF) [file pone.0219865.s004.tif]
